# Rational Design of Enzymatic Electrodes: Impact of Carbon Nanomaterial Types on the Electrode Performance

**DOI:** 10.3390/molecules29102324

**Published:** 2024-05-15

**Authors:** Miroslava Varničić, Tim-Patrick Fellinger, Maria-Magdalena Titirici, Kai Sundmacher, Tanja Vidaković-Koch

**Affiliations:** 1 Max Planck Institute for Dynamics of Complex Technical Systems, Sandtorstr 1, 39106 Magdeburg, Germany; varnicic@ihtm.bg.ac.rs (M.V.); sundmacher@mpi-magdeburg.mpg.de (K.S.); 2 Department of Electrochemistry, Institute of Chemistry, Technology and Metallurgy, National Institute of the Republic of Serbia, University of Belgrade, Njegoševa 12, 11000 Belgrade, Serbia; 3 Division 3.6 Electrochemical Energy Materials, Bundesanstalt für Materialforschung und -Prüfung, Unter den Eichen 44-46, 12203 Berlin, Germany; tim-patrick.fellinger@bam.de; 4 Department of Chemical Engineering, Imperial College London, South Kensington Campus, London SW7, UK; m.titirici@imperial.ac.uk; 5 Process Systems Engineering, Otto-von-Guericke-University Magdeburg, Universitätsplatz 2, 39106 Magdeburg, Germany

**Keywords:** electroenzymatic process, horseradish peroxidase, 3D enzymatic electrodes, peroxide reduction, Vulcan XC72, carbon nanomaterials

## Abstract

This research focuses on the rational design of porous enzymatic electrodes, using horseradish peroxidase (HRP) as a model biocatalyst. Our goal was to identify the main obstacles to maximizing biocatalyst utilization within complex porous structures and to assess the impact of various carbon nanomaterials on electrode performance. We evaluated as-synthesized carbon nanomaterials, such as Carbon Aerogel, Coral Carbon, and Carbon Hollow Spheres, against the commercially available Vulcan XC72 carbon nanomaterial. The 3D electrodes were constructed using gelatin as a binder, which was cross-linked with glutaraldehyde. The bioelectrodes were characterized electrochemically in the absence and presence of 3 mM of hydrogen peroxide. The capacitive behavior observed was in accordance with the BET surface area of the materials under study. The catalytic activity towards hydrogen peroxide reduction was partially linked to the capacitive behavior trend in the absence of hydrogen peroxide. Notably, the Coral Carbon electrode demonstrated large capacitive currents but low catalytic currents, an exception to the observed trend. Microscopic analysis of the electrodes indicated suboptimal gelatin distribution in the Coral Carbon electrode. This study also highlighted the challenges in transferring the preparation procedure from one carbon nanomaterial to another, emphasizing the importance of binder quantity, which appears to depend on particle size and quantity and warrants further studies. Under conditions of the present study, Vulcan XC72 with a catalytic current of ca. 300 µA cm^−2^ in the presence of 3 mM of hydrogen peroxide was found to be the most optimal biocatalyst support.

## 1. Introduction

Bioelectrochemical systems that use enzymes as biocatalysts represent an innovative approach to creating sustainable applications and processes. There has been considerable research into the use of enzymes in biosensing, but their application extends to enzymatic fuel cells and electroenzymatic synthesis as well [1,2,3,4]. These technologies mainly utilize oxidoreductases, which are enzymes that require a pair of substrates for oxidation and reduction reactions. In electroenzymatic processes, one substrate is either replaced by an electrode or supplied/regenerated through electrochemical reactions. This results in two modes of operation: direct and mediated electron transfer. Direct electron transfer (DET) allows enzymes to exchange electrons directly with an electrode without any intermediaries. In contrast, mediated electron transfer (MET) involves a soluble mediator or an enzymatic reaction substrate facilitating the electron transfer between an enzyme and an electrode. Previous publications have highlighted various challenges in developing enzymatic electrodes, with the main issues being low electrode performance and limited durability [4].

To develop enzymatic electrodes with DET that are both highly active and stable, it is crucial to immobilize redox enzymes on electrode surfaces in a way that preserves their activity in a heterogeneous environment. Achieving efficient electron transfer necessitates ensuring optimal contact between the enzymes and the electrode surface, as well as maximizing biocatalyst loading to enhance electrode performance. This approach is expected to increase the current density per unit of geometrical surface area. Electrodes that incorporate these strategies to some degree are known as three-dimensional (3D) electrodes, which have shown significant performance improvements over enzyme-modified planar electrodes termed in the further text as “monolayer” electrodes [5,6].

**Figure 1:**
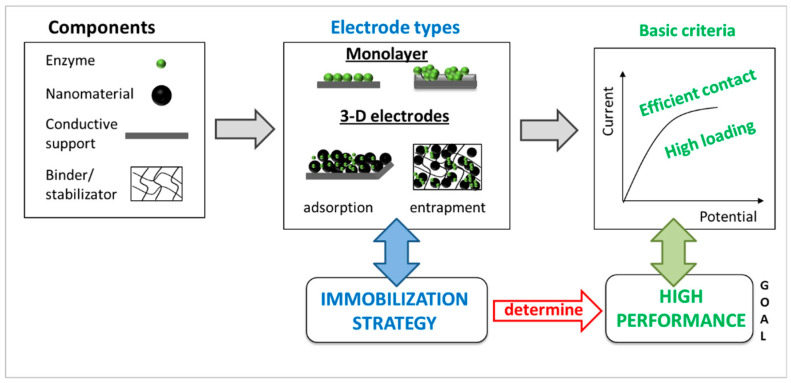
Schematic representation of optimal electrode designing, including different electrode types and criteria for electrode performance with targeted goal.

Three-dimensional enzymatic electrodes are composite materials comprising (1) enzymes serving as biocatalysts, (2) nanomaterials to increase the active surface area and facilitate high enzyme loading, and (3) additives such as hydrogels or cross-linkers to improve electrode stability (Figure 1). The overall performance of these electrodes is influenced by the immobilization method chosen, which determines the spatial organization of the components (nanomaterials, biocatalysts, stabilization additives, and binders) within the 3D structure. It depends further on the type of electron transfer (DET or MET). Below, we focus on DET types of enzymes.

Studies have demonstrated the feasibility of DET for various enzymes, including horseradish peroxidase (HRP), bilirubin oxidase (BOD), and laccase; for other examples, please see [7]. For instance, HRP catalyzes the two-electron reduction of hydrogen peroxide to water, a reaction extensively explored on carbon-based materials ranging from monolayer HRP assemblies on spectroscopic graphite and glassy carbon to 3D electrodes utilizing carbon black materials and carbon nanotubes [2,8,9,10,11]. BOD and laccase catalyze 4-e oxygen reduction to water [12]. DET of these enzymes on different carbon materials such as carbon nanoparticles [13], Carbon Hollow Spheres [14], and Carbon Aerogel [15], but also on metal nanoparticles (e.g., gold) [16], was demonstrated.

Although other materials have also been used (like, as mentioned, gold), carbon-based nanomaterials are among the most commonly used in 3D enzymatic electrodes due to their high conductivity, large surface-to-volume ratio, customizability, and biocompatibility, making them suitable for enhancing reaction kinetics for DET [17].

Despite these advancements, comparing the effects of various preparation procedures and experimental conditions systematically remains a challenge due to their diversity. Consequently, this study aims to systematically evaluate the impact of key parameters, such as the type of carbon nanomaterial on the performance of 3D enzymatic electrodes. We used horseradish peroxidase (HRP) as a model enzyme for the peroxide reduction reaction in conjunction with direct electron transfer. As a reference point, we utilized HRP electrodes prepared by physically adsorbing enzymes onto graphite electrodes, which can be considered a monolayer assembly. Furthermore, we compared novel carbon materials (Carbon Aerogel, Carbon Coral, and Carbon Hollow Spheres) against commercial Vulcan XC72 carbon nanoparticles. In addition to electrochemical characterizations, fluorescence microscopy was employed to gain insights into enzyme dispersion across different electrode preparations.

## 2. Results and Discussion

### 2.1. Monolayer Electrode Design

**Figure 2:**
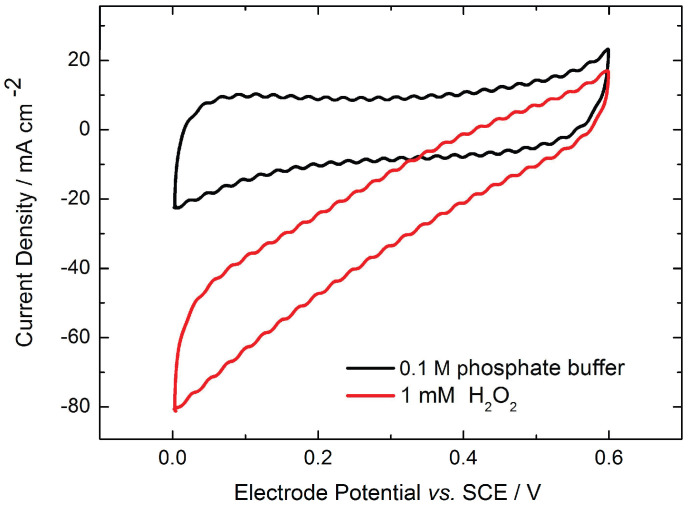
Cyclic voltammetry of HRP adsorbed on the graphite electrode, in 0.1 M phosphate buffer and in the presence of 1 mM hydrogen peroxide; 400 rpm; N_2_ atmosphere; 5 mV s^−1^.

HRP was physically adsorbed on carbon electrodes and electrochemically characterized in the absence and presence of hydrogen peroxide (Figure 2). The cyclic voltammogram of graphite electrodes in phosphate buffer (black line) has a rectangle-like shape, showing no redox process that can be ascribed to DET of the heme group of HRP. This is in accordance with the literature results for other graphite electrodes [18,19].

Upon hydrogen peroxide addition, the reduction currents can be observed (red line). This aligns with the findings in the literature, showing that hydrogen peroxide reduction at the HRP-modified electrodes starts when the electrode is poised at a potential more negative than 0.6 V vs. SCE. The reduction potential is close to the formal potential of Compound-I/II and Compound-II/HRP (Fe^3+^) determined in [20,21], thus confirming peroxide reduction at the HRP electrode.

**Figure 3:**
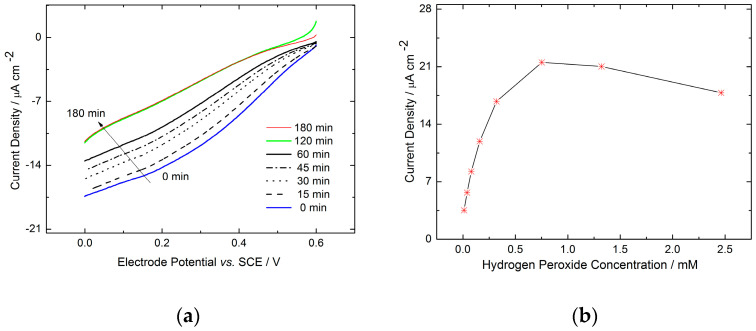
(**a**) Influence of enzyme leaching evaluated in RDE experiments, under constant rotation rate of 400 rpm on HRP-graphite electrode in the presence of 40 μM hydrogen peroxide at pH 6 in 0.1 M phosphate buffer and in N_2_ atmosphere. (**b**) Influence of H_2_O_2_ concentration on the performance of the HRP-graphite electrode at pH 6.00, under constant rotation rate of 400 rpm and at 0.0 vs. SCE in N_2_ atmosphere; current densities in (**b**) are given as absolute values.

One of the disadvantages of the physical adsorption is enzyme leaching. To evaluate this effect after adsorption of HRP at the graphite surface, the electrode performance was evaluated in RDE experiments over time. As can be seen in Figure 3, the current density decreases during the first 2 h at a constant rotation rate of 400 rpm. It becomes stabilized after 2 h at an approximately 30% lower current value compared to the current density at the beginning (0 h). Therefore, to assure stable and reproducible measurements, after enzyme adsorption, the electrode was rotated for 2 h at a constant rotation rate (400 rpm) and then electrochemically characterized. The leaching was also visualized through fluorescence measurements; please refer to our prior work [22]. Only enzyme agglomerates are still present on the surface after accelerated leaching due to electrode rotation. As can be seen (Figure 3b), upon an increase in hydrogen peroxide concentrations, the catalytic currents initially increase. However, at higher concentrations, inhibition by hydrogen peroxide takes places, resulting in current decrease.

### 2.2. Three-Dimensional Electrode Design: Influence of Different Types of Nanomaterials on the Enzymatic Electrode Performance

#### 2.2.1. Material Characterization

**Figure 4:**
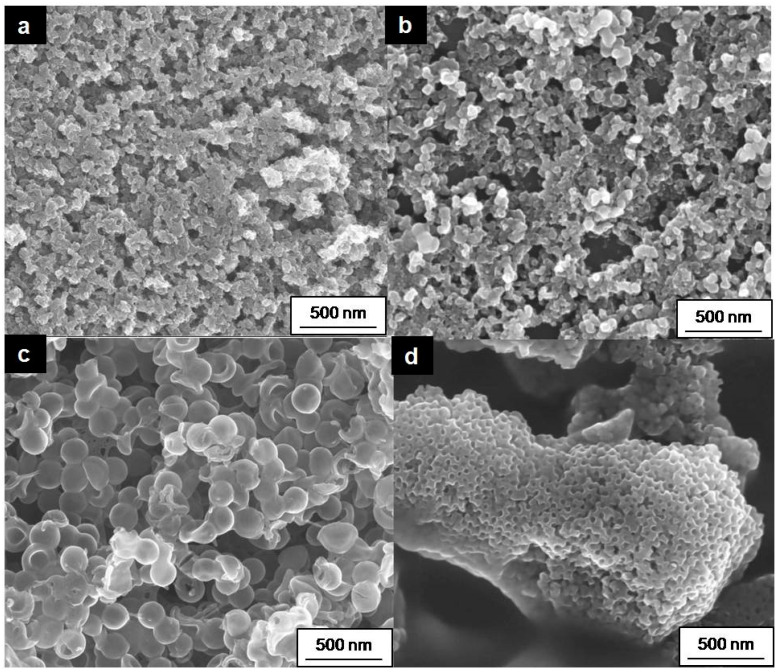
SEM images of (**a**) Carbon Aerogel, (**b**) Vulcan XC72, (**c**) Carbon Hollow Sphere, and (**d**) Coral Carbon.

To examine the impact of nanomaterial type on the performance of enzymatic electrodes, this study focused on two primary types of nanomaterials: (i) nanoparticles and (ii) porous nanomaterials (Table 1). The primary distinction among these nanomaterials lies in their structural differences, as illustrated by the SEM images in Figure 4. Carbon Aerogel, Vulcan XC72, and Carbon Hollow Spheres are spherical particles, with sizes of approximately 14, 80, and 168,nm respectively, and high surface areas of 201, 250, and 377 m^2^/g, respectively. Conversely, Coral Carbon is a mesoporous material with a pore size of 14 nm and a high specific surface area of 528 m^2^/g (as shown in Figure 4d). The utilization of mesoporous materials like Coral Carbon offers the advantage of a porous structure, facilitating well-ordered pores that can be tailored during synthesis to accommodate specific enzyme and substrate sizes. The chosen method for immobilization involves enzyme entrapment via a gelatin procedure (detailed in the experimental Section 3.3), where gelatin plays a crucial role in providing an optimal microenvironment for the proteins and ensuring high electrode stability.

#### 2.2.2. Electrochemical Electrode Characterization

**Figure 5:**
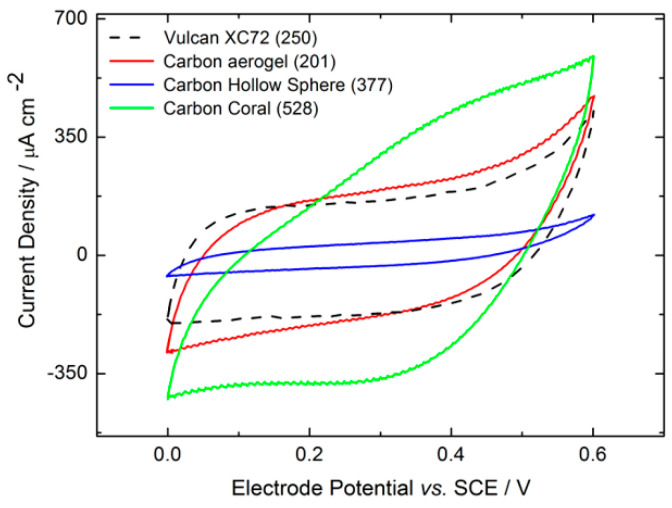
Comparison of cyclic voltammograms for HRP 3D electrodes prepared with various nanomaterials, featuring carbon nanoparticle loadings of 3.5 mg cm^−2^ and HRP loadings of 1.78 mg cm^−2^, conducted in 0.1 M phosphate buffer at pH 6 under N_2_ atmosphere at 400 rpm and a scan rate of 5 mV s^−1^.

Figure 5 presents the cyclic voltammograms (CVs) of enzymatic electrodes incorporating various nanomaterials, recorded in 0.1 M phosphate buffer at a scan rate of 5 mV/s. These CVs typically aim to demonstrate the non-catalytic direct electron transfer of enzymes and provide insights into the surface groups of the carbon materials used. However, the CVs in Figure 5 do not display distinct redox processes associated with either the DET of HRP or the redox-active groups on the carbon materials. This observation is consistent with the findings reported in the existing literature [18]. The lack of discernible peaks, indicative of HRP’s non-catalytic DET, could be attributed to the significant capacitive background signals originating from the carbon nanomaterials. Xia et al. [23] demonstrated that after accounting for background capacitive contributions, reversible peaks associated with the non-catalytic direct electron transfer of HRP were observed at approximately 0.7 V vs. Ag/AgCl in a phosphate buffer at pH 7. The capacitive contributions shown in Figure 5 are linked to the surface area of the carbon materials being examined, serving as indicators of the electrochemically active surface area. Typically, materials with a higher specific surface area are anticipated to exhibit increased capacitive currents and a larger electrochemically active surface area, a notion partially corroborated by the data in Figure 5. Vulcan XC72 and Carbon Aerogel, with similar specific surface areas, demonstrated comparable capacitive currents. Conversely, Coral Carbon, possessing the highest specific surface area, showed the highest capacitive currents. An anomaly was observed with the Carbon Hollow Sphere electrode (377 m^2^/g), which, despite its higher BET surface area, displayed lower capacitive currents than expected when compared to Carbon Aerogel (201 m^2^/g) and Vulcan nanomaterials (250 m^2^/g). This discrepancy might be attributed to the significantly larger particle size of Carbon Hollow Spheres (168 nm) relative to the other carbon nanoparticles (Table 1). Generally, a larger particle size suggests a lower specific surface area for materials of similar densities. However, the lower density of Carbon Hollow Spheres implies a higher number of nanoparticles per unit weight, thus explaining the high specific surface area despite the larger particle size. This also suggests that electrodes made from Carbon Hollow Spheres will have a greater thickness for the same carbon loading compared to those made from other materials, potentially leading to lower electrode utilization and, consequently, lower capacitive currents, as observed.

**Figure 6:**
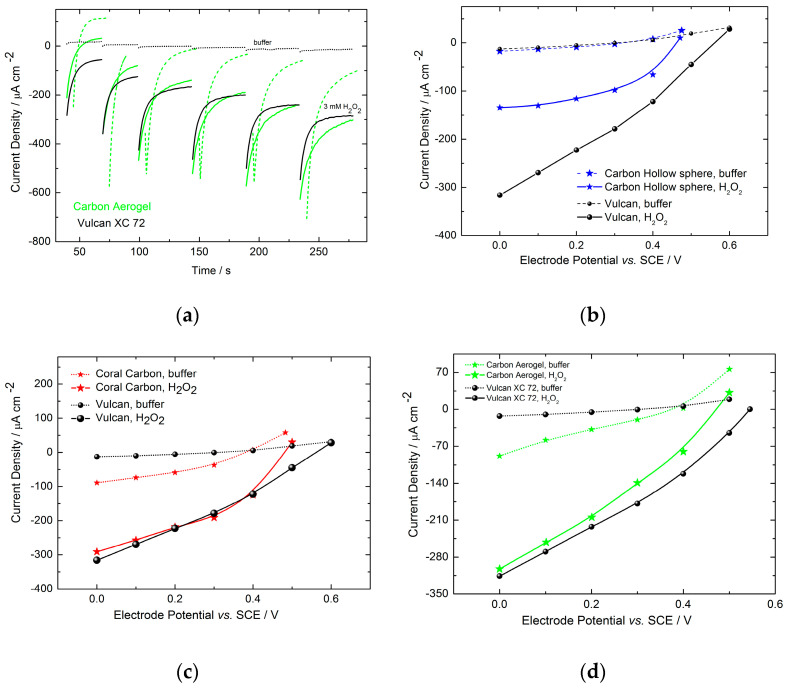
(**a**) Chronoamperometry responses and (**b**–**d**) polarization curves based on currents extracted from corresponding chronoamperometric measurements in 0.1 M phosphate buffer in presence of 3 mM H_2_O_2_, at pH 6 and rotation rate of 400 rpm.

Polarization curves were derived from chronoamperometric measurements, performed with a potential step of 0.1 V, by collecting values after 60 s. Figure 6 showcases chronoamperograms both in the absence and presence of hydrogen peroxide, along with polarizations curves for various materials. All electrodes underwent analysis under identical conditions. The performance of these electrodes relative to Vulcan XC72 is depicted in Figure 6b–d. Notably, both Vulcan XC72 and Carbon Hollow Sphere materials exhibited minimal capacitive currents, indicating that a 60 s timeframe sufficed to achieve a steady-state current response, as shown in Figure 6a,b. Conversely, Carbon Aerogel and Carbon Coral electrodes did not exhibit close-to-zero currents after 60 s in buffer, suggesting that this duration was insufficient for attaining a steady-state response. This could be attributed to the small particle or pore sizes of the Carbon Aerogel and Carbon Coral electrodes, respectively. While the behavior of the Carbon Coral electrode aligns with expectations based on its cyclic voltammetry (CV) profile in Figure 5, the same cannot be said for the Carbon Aerogel electrode, whose CV closely resembles that of Vulcan XC72. Another potential explanation for this phenomenon could be an additional Faradaic process occurring on these materials, possibly involving the oxygen reduction reaction. Although the Carbon Aerogel and Carbon Coral materials used in this study were not evaluated for oxygen reduction reaction, some studies in the literature show oxygen reduction reaction activity of similar materials [24]. Despite careful deaeration of our measurements, the complete absence of dissolved oxygen could not be guaranteed. Given that all experiments were conducted under forced convection (400 rpm), the influence of the oxygen reduction reaction cannot be ruled out and warrants further investigation. Therefore, for comparative results, the catalytic currents of Carbon Aerogel- and Coral Carbon-based electrodes were calculated by subtracting the capacitive currents in phosphate buffer from the total current obtained in the presence of hydrogen peroxide.

**Figure 7:**
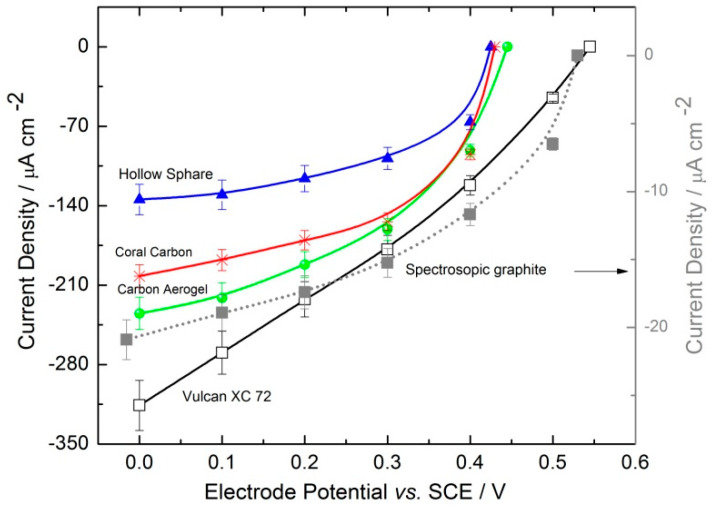
Comparison of the activities of various 3D HRP electrodes in the presence of 3 mM H_2_O_2_ in a 0.1 M phosphate buffer under an N_2_ atmosphere, at a pH of 6 and a rotation rate of 400 rpm. The activity of an HRP-modified graphite electrode was also evaluated under similar conditions, but with a reduced H_2_O_2_ concentration of 1 mM.

Figure 7 compares the catalytic activities of various 3D enzyme-based electrodes in the presence of 3 mM of hydrogen peroxide. As benchmarks, both a Vulcan XC72-based electrode and a flat graphite electrode modified with a physically adsorbed HRP layer were used. The latter was tested at a lower hydrogen peroxide concentration due to enzyme inhibition by the substrate at higher concentrations, as previously demonstrated (Figure 3). Electrodes based on carbon nanomaterials also exhibit inhibition by hydrogen peroxide, but at higher concentrations. This phenomenon was investigated using Vulcan XC72 as an example in our prior work [22,25]. The high surface area of carbon nanomaterials facilitates the immobilization of a greater amount of biocatalysts compared to flat electrodes, allowing for the use of higher peroxide concentrations (in the mM range). As shown in Figure 7, the electrode based on Vulcan XC72 surpasses the performance of all other tested materials, achieving a current density of approximately 300 µA cm^−2^ at 0.0 V vs. SCE. Cyclic voltammograms conducted without hydrogen peroxide (Figure 5) reveal that Vulcan XC72 and Carbon Aerogel possess similar electrochemically active surface areas. However, their differing efficacies in reducing hydrogen peroxide suggest variations in surface utilization and the percentages of active biocatalysts with optimal orientation within the catalyst layer. Despite the Coral Carbon electrode having the largest electrochemically active surface area among the materials tested, its performance (approximately 200 µA cm^−2^) falls short of that achieved by Vulcan XC72 (approximately 300 µA cm^−2^) and Carbon Aerogel (approximately 240 µA cm^−2^). Moreover, the onset potential for the Vulcan XC72 electrode is 0.57 V vs. SCE, which is 0.12 V more positive than that of the other nanomaterials (approximately 0.45 V), indicating enhanced direct electron transfer at Vulcan XC72. This onset potential of 0.57 V vs. SCE is similar to that observed for the HRP-modified graphite electrode (Figure 2). The performance metrics for Vulcan XC72 align well with published results for Ketjen Black modified glassy carbon electrodes [23]. The values observed for other carbon nanomaterials are close to the literature values reported for multi-walled carbon nanotubes: ca. 0.45 V vs. SCE at pH 7.40 [26]. Three-dimensional electrodes based on Vulcan XC72 exhibit approximately 14 times higher catalytic currents compared to HRP-modified graphite electrodes (referred to as “monolayer assembly”), while the difference in active surface area (determined through cyclic voltammetry in the absence of hydrogen peroxide) is about 17 times greater. This suggests that the observed enhancement primarily stems from an increase in active surface area, thereby facilitating greater enzyme loading. Furthermore, our previous work [27,28] demonstrated that mass transport limitations within porous catalyst layers lead to reduced enzyme utilization, in contrast to monolayer assemblies where enzymes are uniformly accessible to the substrate. The performances of various electrodes are summarized and compared with the literature results for similar 3D HRP electrodes (Table 2), focusing on non-chemically modified carbon nanomaterials and results at the same hydrogen peroxide concentration (3 mM). In Table 2, additional details on immobilization strategies, enzymes, and carbon nanomaterial loadings are also provided. The Ketjen Black electrode, with a limiting current density of approximately 7.5 mA cm^−2^, demonstrated the best performance [23]. This electrode features the highest BET surface area of 800 m^2^ g^−1^, which correlates with improved performance due to the increased surface area, as already discussed. Furthermore, the method of enzyme immobilization significantly impacts performance. For instance, 3D HRP electrodes using the same type of carbon nanomaterial (Vulcan XC) but a different immobilization strategy showed markedly different performances. The electrode with physically adsorbed enzymes [22] exhibited a performance roughly three times higher than that of the 3D HRP electrode prepared by entrapment using a gelatin binder and GA for cross-linking in this work. This suggests that adsorption is a more effective immobilization strategy than entrapment for enzymatic electrodes with direct electron transfer. Additionally, the role of mass transport cannot be ignored. The enzymatic reduction of hydrogen peroxide by HRP is dependent on mass transport [27], with improved conditions (e.g., higher rotation rates) enhancing electrode performance. The Ketjen Black electrode [23] was tested at 4000 rpm, in contrast to Vulcan XC [22] and other electrodes in this study, which were tested at 400 rpm. Moreover, enzyme loadings are crucial; Ketjen Black benefits from higher enzyme loadings than Vulcan XC, leading to higher current densities. However, as shown by the MWCNT [26] example, high enzyme loadings alone are insufficient if mass transport conditions are suboptimal. The impact of mass transport on the concentration profiles of 3D HRP electrodes was theoretically examined in our previous publication [27], revealing that concentration profiles depend on rotation rate, as well as the porosity and thickness of the 3D porous layer. Under certain conditions, large portions of the catalyst layers may remain unutilized for the reaction due to hydrogen peroxide depletion. To further investigate the effects of electrode morphology and enzyme distribution across different materials, microscopy characterizations were performed.

#### 2.2.3. Investigation of the Electrode Cross-Sections

**Figure 8:**
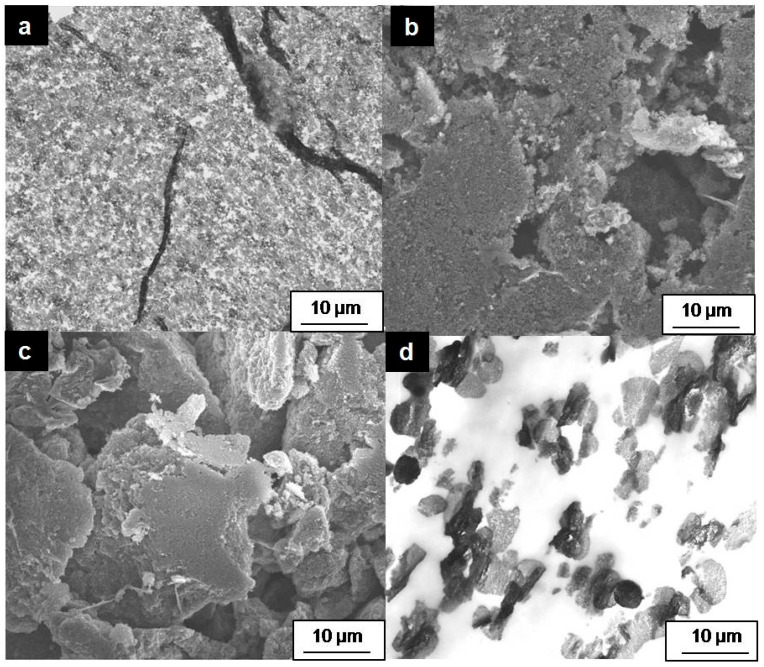
SEM images of electrode cross-sections: (**a**) Carbon Aerogel nanomaterial, (**b**) Vulcan XC72, (**c**) Carbon Hollow Sphere, and (**d**) Coral Carbon.

The SEM characterizations of electrode cross-sections are depicted in Figure 8. The Vulcan XC electrode exhibits a very homogeneous structure, albeit with some large cracks. The structures of Carbon Aerogel and Carbon Hollow Spheres, despite the significant differences between the two materials, appear relatively similar. In contrast, the structure of the Coral Carbon electrode is markedly different. Unlike the other materials, large portions of the Coral Carbon are obscured by gelatin (indicated by white areas), suggesting that the gelatin quantity used for the preparation of this electrode was overestimated. Coral Carbon possesses a very large specific surface area, primarily due to the presence of small pores, while the material consists of large monoliths. The results suggest that the gelatin could not penetrate the small pores and instead formed thick films around the monoliths. This phenomenon could account for the lower catalytic activity observed in Coral Carbon electrodes, as the thick gelatin layer impedes the mass transport of hydrogen peroxide to the catalytic sites. The gelatin distribution appears to be most optimal in Vulcan XC72. Carbon Aerogel and Carbon Hollow Spheres exhibit some uniform regions, but there are indications that some macropores were created during the preparation process. This might suggest that the gelatin quantity was underestimated in these instances, leading to the formation of larger agglomerates with gelatin films surrounding them. The findings further imply that the preparation procedure developed for one material cannot be directly applied to other carbon nanomaterials. It highlights the necessity of adjusting the binder amount (gelatin in this case) according to the particle size and quantity.

### 2.3. Characterization of the Enzyme Distribution

Fluorescence microscopy was utilized to analyze the distribution of enzymes within 3D electrodes. To facilitate this, samples were prepared on glass supports and the enzymes were labeled with a fluorescent dye for visualization purposes (refer to the experimental section for details). The electrode preparation process included cross-linking with glutaraldehyde; therefore, two distinct sample types were prepared. The first sample type involved a catalyst ink comprising selected carbon nanomaterials, enzymes, and gelatin. These samples are further referred to as non-cross-linked. The component ratios mirrored those used in the fabrication of 3D enzymatic electrodes. However, the applied ink featured a reduced thickness compared to that of the 3D electrodes, resulting in a film that was essentially 2D. The second sample type was prepared similarly to the first, with the addition of a cross-linking step, and is referred to further as the cross-linked sample. This cross-linking process is known to stabilize enzymatic electrodes but also reduces enzyme activity, as demonstrated in our previous work [22]. For fluorescence microscopy characterizations, two types of carbon nanomaterials were employed: Vulcan XC72 and Coral Carbon.

**Figure 9:**
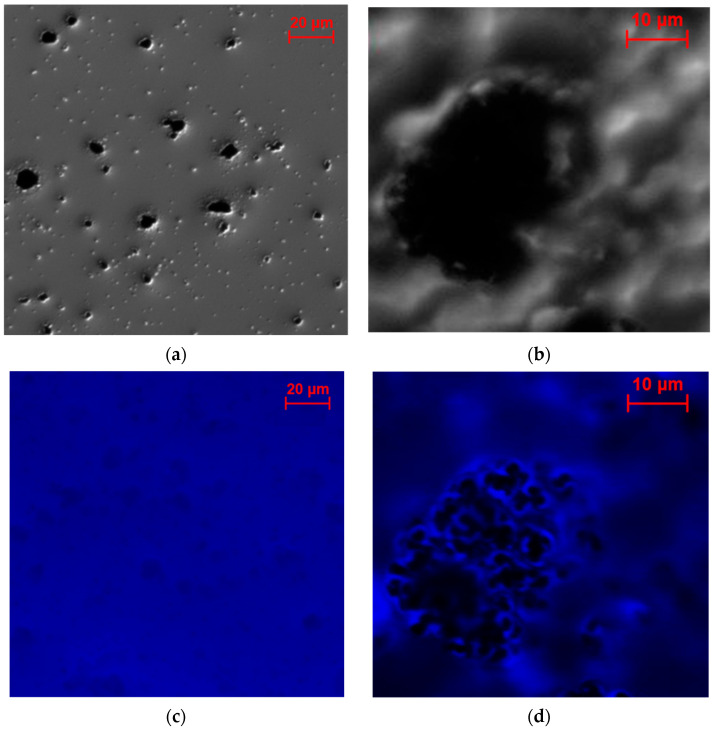
Optical microscopy of non-cross-linked samples on glass supports: (**a**) Vulcan XC72, (**b**) Coral Carbon, and corresponding fluorescence microscopy images of (**c**) Vulcan XC72 and (**d**) Coral Carbon.

Figure 9 displays the optical and fluorescence microscopy micrographs of non-cross-linked samples. For Vulcan XC72, small agglomerates ranging in size from 5 to 10 μm are visible, whereas Coral Carbon exhibits a single large agglomerate measuring 10 × 30 μm (Figure 9a,b). The blurred area in Figure 9b results from the focus being on the agglomerate, which was positioned at a higher level than its surroundings. These samples were further examined using fluorescent microscopy to assess enzyme distribution (Figure 9c,d). It is evident that Vulcan XC72 agglomerates are entirely coated with labeled enzymes, while the large parts of Coral Carbon are not covered by enzymes.

**Figure 10:**
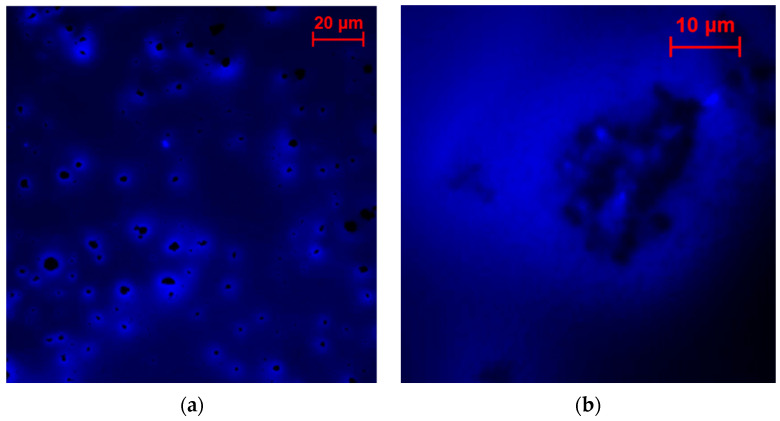
Fluorescence micrographs showing influence of cross-linking on the enzyme distribution at different nanomaterial surfaces: (**a**) Vulcan XC72 and (**b**) Coral Carbon.

The fluorescence microscopy micrographs post-glutaraldehyde cross-linking are presented in Figure 10. For Vulcan XC72, the cross-linking appears to cause enzyme concentration around Vulcan XC72 agglomerates, suggesting a reduction in enzyme–support contact, potentially leading to decreased activity. A similar effect was previously observed for HRP-modified graphite electrodes after cross-linking [22]. In the case of Coral Carbon, a significant portion of enzymes seems to remain within the gelatin matrix, rendering them inaccessible for direct electron transfer on the surface. This phenomenon likely accounts for the low activity observed with this carbon nanomaterial.

## 3. Materials and Methods

### 3.1. Chemicals and Materials

Horseradish peroxidase (EC 1.11.1.7, HRP) from Amorica rusticana was provided by Serva Electrophoresis GmbH, Heidelberg, Germany. Hydrogen peroxide (H_2_O_2_, 30 wt%) and gelatin for microbiology were supplied from Merck, Darmstadt, Germany. The hydrogen peroxide solution, with a concentration of 3 wt%, was prepared daily by dilution of 30 wt% hydrogen peroxide. Spectroscopically pure carbon rods (graphite, SPG) with impurities equal to or less than 2 ppm were supplied by Ted Pella, 330 Inc., Redding, CA, USA. The Vulcan XC72R nanomaterial for the preparation of porous enzymatic electrodes was provided by Cabot Corporation, Boston, MA, USA. Protein labeling for fluorescence measurements was achieved using DyeLight 350NHS ester dye by Thermo Scientific, Waltham, MA, USA with an excitation wavelength of 353 nm and an emission wavelength of 432 nm. Potassium dihydrogenphosphate was provided by Carl Roth GmbH&Co.KG, Karlsruhe, Germany. Sodium phosphate dibasic was purchased from Sigma-Aldrich, St. Louis, MO, USA. All solutions were prepared using ultrapure water from Millipore and all chemicals were of analytical reagent grade.

### 3.2. Carbon Nanomaterial Synthesis

Carbon Hollow Spheres:

An aqueous dispersion of polystyrene particles of 100 nm (3 wt%) was mixed with D-glucose acting as the carbon precursor. The reaction mixture was heated at 200 °C for 20 h. After reaction, the carbonaceous product was filtered off, followed by washing with excess water and drying under vacuum. To remove the polymer template and to graphitize the carbonaceous shell, the composite material was than heated to the desired temperature above the template decomposition point (e.g., >500 °C). Here, the materials were carbonized in a tube furnace under N_2_ at 700 °C for 4 h.

Coral Carbon:

In the synthesis of Coral Carbon, 0.25 g of Pluronic F127 (Mw = 12 500, EO106-PO70-EO106) and 2.4 g of D-fructose were dissolved in 8 mL of distilled water. A total of 2 mL of the polystyrene latex PSL dispersion (5 wt%) was then added followed by vigorous stirring at room temperature for 10 min. The system was heat-treated either at 130 °C for 120 h or at 180 °C for 48 h in a stainless autoclave (Paar, Acid digestion vessel). After reaction, the obtained carbon monolith was washed with excess distilled water and ethanol and dried at 65 °C overnight. The polymer templates were removed by calcination under N_2_ either at 550 °C or at 900 °C for 4 h.

Carbon Aerogel:

Carbon Aerogel was synthesized by the hydrothermal carbonization (HTC) method at 200 °C for 8 h in a Teflon-lined 45 mL Anton-Parr pressure vessel equipped with a glass vial. A total of 500 mg of borax and 20 mL of 20 wt% aqueous D-glucose solution were mixed, and the gas-tight closed pressure vessel was transferred to the pre-heated oven for hydrothermal carbonization under self-generated pressure. After HTC, the vessel was quickly cooled down in a room-temperature water bath. A light-brown-colored and mechanically stable monolith was recovered, extracted three times with deionized water, and further washed with abs. ethanol (3 times, 50 mL). After purification and changing the solvent back to water, the sample was freeze-dried and transferred within an alumina boat into an inert gas Nabertherm quartz tube furnace. The sample was carbonized in a nitrogen atmosphere with a heating ramp of 10 K/min to 900 °C and held for 1 h before it was allowed to cool down to room temperature. All used chemicals were purchased from Sigma-Aldrich and used as received.

### 3.3. Enzymatic Electrode Preparations

In studies involving monolayer electrodes, graphite served as the substrate for enzyme adsorption. Prior to enzyme modification, the graphite surface was first polished using fine emery paper (grades P500 and P1000), followed by additional polishing with standard white paper to enhance smoothness. The adsorption of horseradish peroxidase (HRP) was achieved by applying 50 µL of an HRP solution (concentration: 6 mg/mL, pH 6.00) onto the graphite disk and allowing it to adsorb for 2 h at room temperature. Subsequently, the disk were rinsed with phosphate-buffered solution before being utilized for experimental measurements. The geometrical electrode’s surface area designated for HRP adsorption and subsequent electrochemical analysis was 0.28 cm^2^.

**Figure 11:**
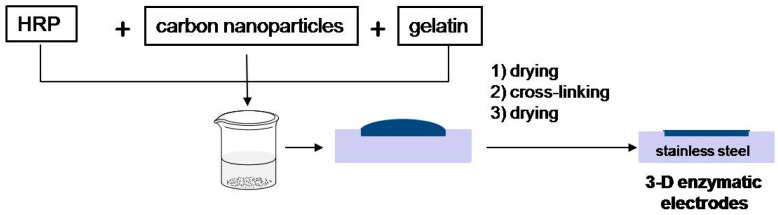
Enzymatic electrode preparation procedure, where carbon nanoparticles are the tested nanomaterials shown in Table 1.

Porous enzymatic electrodes were prepared by the following steps (please see Figure 11): A gelatin solution (2 wt% in ultrapure water) was prepared by heating the solution up to 37 °C. In the next step, 20 mg of respective nanomaterial (Vulcan XC72 or synthesized carbon nanomaterials) and 10 mg of HRP were suspended in 1 mL of gelatin at 37 °C, and 50 µL of the described ink was cast into the stainless steel disks (11 mm diameter) that were used as mechanical and electrical support. The electrodes were subsequently dried at room temperature and cross-linked. The cross-linking procedure consisted of the following: prepared enzymatic electrodes were dipped in 5 wt% glutaraldehyde solution and left for 1 min. Then, electrodes were washed with deionized water and were stored in a refrigerator at a temperature of −18 °C.

For fluorescent microscopic studies, the enzyme was labeled in the following way: HRP solution (pH 7.00, 2 mg mL^−1^) was mixed with fluorescence dye dissolved in dimethylformamide (DMF) and left for 1 h at room temperature. Afterwards, the excess of non-reacted dye was removed by dialysis for 4 h using three dialysis buffer changes. The labeled enzymes were stored at 4 °C. For obtaining fluorescence microscopy images with carbon nanomaterial, 1 mg of carbon material (Vulcan or synthesized nanomaterial) was added to the HRP-labeled solution (2 mg mL^−1^, pH 7.00). The mixture was stirred and cast on the glass plate to be used for microscopy investigation.

### 3.4. Measurements

The electrochemical experiments were carried out in the three-electrode configuration with a rotating disk electrode (RDE, Radiometer Analytical, model ED101). The enzymatic electrodes prepared with different carbon materials or HRP-modified graphite electrodes were used as the working electrode, while the reference and counter electrodes were saturated calomel (SCE) and Pt, respectively. Steady-state polarization curves were obtained by extracting the current values from chronopotentiostatic measurements after 60 s at constant potential values. In all measurements, the electrolyte was 0.1 M phosphate buffer with pH 6.0. All electrochemical experiments were carried out in a N_2_ atmosphere and at 400 rpm. Electrochemical experiments were performed using an Autolab potentiostat (PGSTAT302, Eco Chemie, Utrecht, The Netherlands).

The specific surface areas of carbon nanomaterials were measured using a Quantachrome Quadrasorb physisorption porosimeter, employing N_2_ adsorption–desorption isotherms at a temperature of 77 K. The specific surface area values were calculated from these isotherms by applying the BET method within the relevant relative pressure range, using the Quantachrome AsiQ software, version 3.0.

Particle sizes were determined be scanning electron microscopy using a LEO 1550-Gemini system.

Fluorescence microscopy was performed with an Imager M1 Microscope, Carl Zeiss, Oberkochen, Germany. The objective was EC Plan Neofluar, and a filter set with excitation 365, beamer splitter 395, and emission 445/50 was used. Cross-sectional scanning electron microscopy of electrodes was performed using XL30 FEG (FEI Company, Hillsboro, OR, USA).

## 4. Conclusions

In this study, we investigated the impact of different types of carbon nanomaterials on the catalytic activity of 3D enzymatic electrodes. We compared as-synthesized carbon nanomaterials, including Carbon Aerogel, Coral Carbon, and Carbon Hollow Spheres, with the commercial Vulcan XC72 carbon nanomaterial. The 3D electrodes were fabricated using gelatin as a binder and cross-linked with glutaraldehyde. In the absence of hydrogen peroxide, none of the electrodes exhibited any redox processes related to the adsorbed enzymes. The capacitive behavior observed was primarily attributed to the BET surface area of the materials under study. The catalytic activity towards hydrogen peroxide reduction was partially linked to the capacitive behavior in the absence of hydrogen peroxide. Notably, the Coral Carbon electrode demonstrated large capacitive currents but low catalytic currents, an exception to the observed trend. Microscopic analysis of the electrodes indicated suboptimal gelatin distribution in the Coral Carbon electrode. Fluorescence microscopy further showed that a significant portion of enzymes was also buried within the gelatin film and detached from the electrode surface, explaining the lower activity of Coral Carbon compared to Vulcan XC72 and Carbon Aerogel. This study also highlighted the challenges in transferring the preparation procedure from one carbon nanomaterial to another, emphasizing the importance of binder quantity, which appears to depend on particle size and quantity and warrants further studies. Under the conditions of the present study, Vulcan XC72 was found to be most optimal biocatalyst support. However, the findings suggest that using gelatin as a binder for cross-linking is not optimal for 3D electrodes with direct electron transfer due to the enzyme agglomeration within the electrode porous structure.

## Figures and Tables

**Table 1:** Overview of different carbon materials and their characteristics.

Type of Material	Name	BET, m^2^ g^−1^	Particle/Pore Size, nm
Particles	Carbon Aerogel	201	14/-
	Vulcan XC72	250	80/-
	Carbon Hollow Sphere	377	168/-
Porous	Coral Carbon	528	-/14

**Table 2:** Performance comparison of the 3D HRP electrodes employing DET, at 3 mM H_2_O_2_ in 0.1 M phosphate buffer.

Carbon Nanomaterial	BET Surface Area/m^2^ g^−1^	Immobilization Procedure	Enzyme and Carbon Loadings, mg cm^−2^	Scan Rate/mV s^−1^	Rotation Rate/rpm	*j*_max/_ mA cm^−2^	Ref.
MWCNT	250–300 [28]	Adsorption	2.8 and 0.7	10	no	0.3 *	[26]
KB	800 [29]	Adsorption/cross-linking with GA	1.4 and N.A.	5	4000	ca. 7.5 *	[23]
Vulcan XC72	250	Adsorption	0.31 and 1	SS	400	1.0	[22]
Vulcan XC72	250	Entrapment	0.52 and 1.05	SS	400	0.31	This work
Carbon Aerogel	201	Entrapment	0.52 and 1.05	SS	400	0.24	This work
Coral Carbon	528	Entrapment	0.52 and 1.05	SS	400	0.21	This work
Carbon Hollow Spheres	377	Entrapment	0.52 and 1.05	SS	400	0.14	This work

* pH 7.

## Data Availability

The original data presented in this study are openly available in Edmond at [https://doi.org/10.17617/3.J3MRIP].
